# Experiences of advanced dementia care in seven European countries: implications for educating the workforce

**DOI:** 10.1080/16549716.2018.1478686

**Published:** 2018-08-13

**Authors:** Manuel Lillo-Crespo, Jorge Riquelme, Rhoda Macrae, Wilson De Abreu, Elizabeth Hanson, Iva Holmerova, Mª José Cabañero, Rosario Ferrer, Debbie Tolson

**Affiliations:** a Faculty of Health Sciences, University of Alicante, Alicante, Spain; b School of Health Nursing and Midwifery, University of the West of Scotland, Hamilton, Scotland; c Universidade do Porto Faculdade de Medicina, Escola superior de Enfermagem do Porto, Portugal; d Health and Caring Sciences, Linnaeus University, Växjö, Sweden; e Faculty of Medicine, Univerzita Karlova v Praze, Czech Republic

**Keywords:** Dementia, Alzheimer disease, case study, caregivers, quality improvement

## Abstract

**Background**: There is a paucity of robust research concerning the care experiences of peoplewith advanced dementia within Europe. It is essential to understand these experiences if weare to address care inequalities and create impactful dementia policies to improve servicesthat support individuals and enable family caring.

**Objectives**: To identify the strengths and weaknesses in daily life perceived by people with dementia and family caring across Europe by exemplifying experiences and the range of typical care settings for advanced dementia care in seven partner countries.

**Methods**: Twenty two in-depth qualitative case studies were completed in seven European countries across a range of care settings considered typical within that country. Narrative accounts of care illuminated a unique set of experiences and highlighted what was working well (strengths or positive aspects) and not so well (weaknesses or negative aspects) for people with advanced dementia and family caring. A constant comparative method of analysis through thematic synthesis was used to identify the common themes.

**Results**: Eight key themes were identified; Early diagnosis, good coordination between service providers, future planning, support and education for carers, enabling the person with dementia to live thebest life possible and education on advanced dementia for professional and family caregiverswere all significant and recurring issues considered important for care experiences to bepositive.

**Conclusion**: People with advanced dementia may have limited opportunities for self-realization and become increasingly reliant on the support of others to maximize their health and well-being. Careful attention must be given to their psychosocial well-being, living environment and family caring to enable them to live the best life possible. Building on what the case studies tell us about what works well, we discuss the potential for integrating the findings into interprofesional learning solutions for the professional workforce across Europe to champion practice-based change.

## Background

The predicted rise in the ageing population and the incidence of age-related diseases, including dementia, is a major public health concern [1]. Over the coming decades, Alzheimer’s disease and other dementias will become the primary public-health concern, with the forecast that globally there will be 100 million people with dementia by 2050 [2] and 14 million of these will be in Europe [3]. Dementia has been a long-standing concern in Europe, where major research efforts have focused on understanding the pathogenesis and epidemiology of dementia, and comparing this data outcomes from the USA. Curiously, there has been little attention on the experiences of people with advanced dementia which is associated with inherent healthcare complexities and variations in Health Care System models across Europe. As the last *Dementia in Europe Yearbook 201* *3* stated, the needs and experiences of receiving and living with a diagnosis of dementia are unique and complex, though the elements of the care pathways related to institutional care and end-of-life care have not been reviewed in depth [4]. Governments have been called to commit to providing good quality care and services for people with dementia [5]. Countries have been asked to build and share solutions to deliver services across the continuum of care [6] and to prioritize and improve the training and development of the dementia workforce [5,7]

This study was completed as part of a larger European Union Erasmus+ funded project, which aligns to the European Higher Education Modernization Agenda [8]. The dementia Palliare Project aimed to develop a range of social participatory and practice-based learning resources to equip qualified practitioners from all health and social care disciplines to champion improvements to advanced dementia care in their workplace [9]. A previous study conducted in eight European countries in 2011 by Karlsson et al. and published in 2014 [10] investigated people with dementia and their informal caregivers’ views of inter-sectoral information, communication and collaboration throughout the trajectory of dementia care. That study used focus groups and highlighted as the core finding that information, communication and collaboration were to be focused on the people with dementia and the informal caregivers; entering into the trajectory of the disease and its consequences was addressed as an important point of departure; the relation to professional care required establishing a trusting relationship, tailor-made intervention and a single person or organization to contact; and professional knowledge and commitment, variation in service and care adapted to needs were important. Although population-based studies in Europe have contributed to the understanding of the clinic-pathologic basis of dementia from an epidemiological perspective, particularly in the oldest age groups, this paper brings to prominence the experience of care for those living with advanced dementia, for their family members and the paid carers of the person being cared for. Therefore, the study aim was to identify the positive aspects and negative aspects from the perspective of those who live with dementia (understood as those affected by dementia and those involved in dementia caring experience). These illuminate the different policy and cultural context of caring and being cared for in Europe, illuminating the need for evidence-informed advanced dementia care. They also highlight the importance of increasing access to high-quality education on advanced dementia care that could help bridge the variations in care and education across Europe.

## Methods

### Study design and procedures

This paper presents data from 22 case studies that explored the experience of advanced dementia care in the 7 countries involved: Scotland (overall project lead), Spain (case study lead), Sweden, Finland, Slovenia, Czech Republic, and Portugal. Figure 2 below shows the total number and percentage of people with dementia in the population of each participating country.

**Figure 1.**
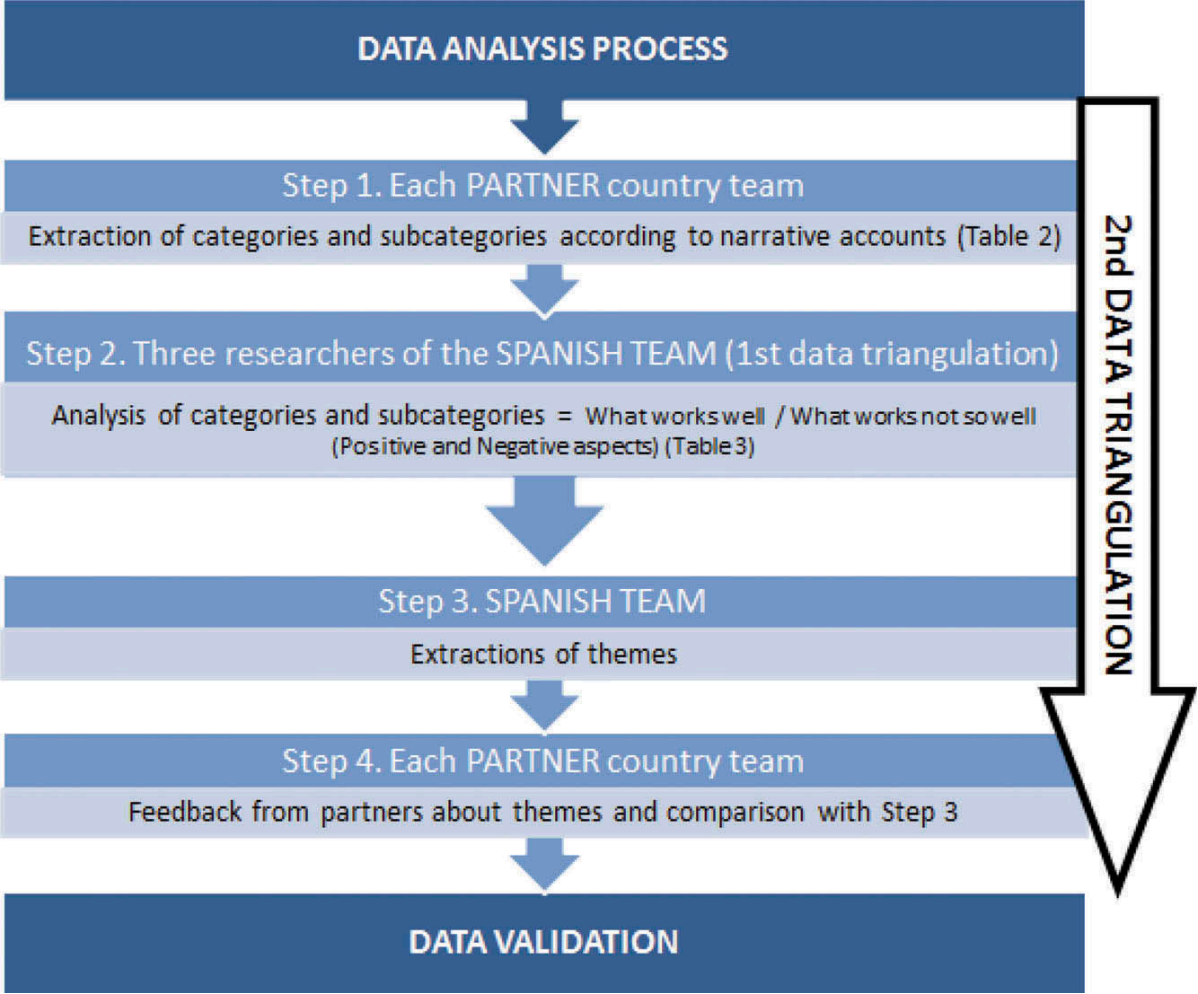
Four-step and double triangulation data analysis procedure.

**Figure 2.**
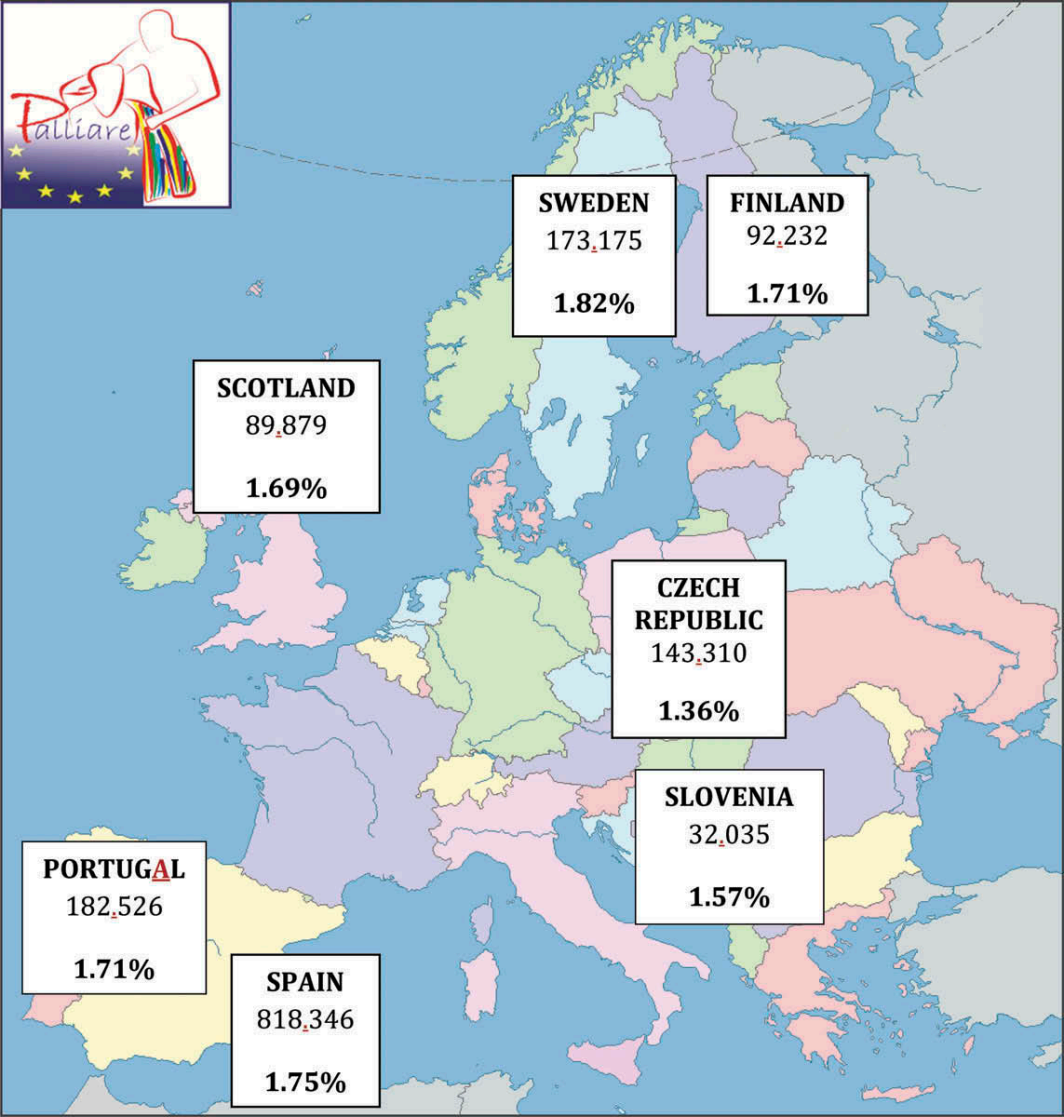
Countries, number and percentage of dementia population (Alzheimer Europe 2014).

The case studies exemplify the experiences of people with advanced dementia, a family member and paid carer in a range of care settings that care for people with advanced dementia. They illuminate a unique set of experiences and are set within particular care environments that are considered typical within each partner country. Six teams constructed three ‘typical’ in-depth case studies each; the Portuguese team undertook four case studies. A case study design was selected according to Yin (2003) as: (a) the focus of our study was to answer ‘how’ and ‘why’ questions about the care experience in dementia; (b) the behaviour of those involved in the study could not be manipulated; (c) we wanted to cover contextual conditions because we believed they were relevant to the phenomenon under study; (d) the boundaries were not clear between the phenomenon and the context. It would have been impossible for this study to have a true picture of the phenomenon studied without considering the context within which it occurred.

Moreover, the case study method is a useful strategy for the investigation of a complex phenomenon [11] such as the experience of advanced dementia care in different care contexts, geographical areas and cultures. The cases considered as ‘typical’ were selected by each partner team composed by recognized experts in the field of dementia care in each partner country and contrasted with previous published information regarding dementia demographics, healthcare systems and services. Each case was considered as a unity of subjects that could offer a usual revealing set of circumstances within each context. Therefore, the case selection was based on representativeness according to researchers’ in-depth local knowledge who were in a position to ‘soak and poke’, as Fenno (1986) stated, and thereby to offer reasoned lines of explanation based on this rich knowledge of setting and circumstances [12]. The type of case study selected was a multiple case study defined by Yin (2003) as the one that enables researchers to explore differences within and between cases in distinct contexts [13]. Because comparisons are drawn in this type, it is imperative that the cases are chosen carefully so that the researcher can predict similar results across cases, or predict results.

Each team applied to the relevant ethical committees in their own countries and sought approval to conduct the cases studies. Participants were selected and contacted by each partner team’s leads that were in contact with the health institutions and associations in each partner country. In Sweden and Slovenia, ethical approval to talk directly to people with dementia could not be sought within the timescales of the project so the professional workforce that had worked with or undertaken research on people with advanced dementia constituted the case study informants in these countries. The inclusion criteria were that the person with dementia participating could demonstrate that they had been diagnosed with advanced dementia, defined as the stage that goes from the middle to the severe phase of the disease having a clinical diagnosis, as shown in Table 1. At least one of the three people with dementia within each partner country had retained verbal skills. The inclusion of family members was based on them having been or still being the main carer. The inclusion of paid carers was on the basis that they had supported the person with dementia for more than six months. The study was conducted between January and April 2015. A total of 56 interviews was conducted; 21 of these were with people with dementia, 23 were with family members and 12 with paid staff from healthcare and social fieldwork.

**Table 1.** Demographics and baseline characteristics per country and case.

Country	Case	People interviewed (number, gender and role)	Gender of the person with advanced dementia	Age of the person with advanced dementia	Years since diagnosis of advanced dementia	Current setting of the person with advanced dementia	Main caregiver of the person with adavanced dementia	Family support (involvement level)	Communication skills of the person with dementia
Scotland	1	1-Pwd (F) 2-Husband (M) 3-Nurse (F)	Female	N/A	1	Own home	Husband	Direct	Yes
	2	1-Pwd (M) 2-Daughter (F) 3-Healthcare staff (M)	Male	N/A	1	Own home/hospital ward	Daughter	Direct	Yes
	3	1-Pwd (F) 2-Nurse (F)	Female	N/A	4	Nursing home	Healthcare staff	Indirect	Yes
Slovenia	1	1-Pwd (F) 2-Daughter (F) 3-Nurse (F)	Female	N/A	N/A	Own home	Daughter	Direct	Yes
	2	1-Pwd (F) 2-Social worker (M) 3-Nurse (F)	Female	79	2	Nursing home	Healthcare staff	Indirect	YES
	3	1-Nurse (F)	Female	-	-	-	-	-	-
Portugal	1	1-Pwd (F) 2-Husband (M) 3-Nurse (F)	Female	83	3	Own home	Husband	Direct	No
	2	1-Pwd (M) 2-Wife (F) 3-Daughter (F) 4-Nurse (F)	Male	85	5	Own home	Wife	Direct	Yes
	3	1-Pwd (M) 2-Wife (F) 3-Nurse (F)	Male	65	2	Own home/day centre	Wife	Direct	Yes
	4	1-Pwd (M) 2-Daughter (F) 3- Nurse (F)	Male	83	1	Relative’s home/day centre	Daughter	Direct	Yes
Finland	1	1-Pwd (M) 2-Wife (F)	Male	71	6	Own home/day centre	Wife	Direct	Yes
	2	1-Pwd (F) 2-Daughter (F)	Female	80	2	Dementia care home	Healthcare staff	Indirect	Yes
	3	1-Pwd (F) 2-Daughter (F)	Female	88	2	Own home	Healthcare staff	Indirect	Yes
Sweden	1	1-Pwd (F) 2-Daughter (F)	Female	71	6	Nursing home	Family and healthcare staff	Indirect	Yes
	2	1-Pwd (F) 2-Husband (M)	Female	78	N/A	Nursing home	Healthcare staff	Indirect	No
	3	1-Pwd (M) 2-Wife (F)	Male	80	N/A	Nursing home	Healthcare staff	Indirect	No
Czech republic	1	1-Pwd (F) 2-Daughter (F)	Female	90	17	Own home/hospital ward	Daughter	Direct	No
	2	1-Pwd (F) 2-Daughter in law (F) 3- Son (M)	Female	81	7	Own home	Daughter in law	Direct	No
	3	1-Pwd (F) 2- Daughter (F)	Female	86	12	Own home	Daughter	Direct	No
Spain	1	1-Pwd (F) 2-Daughter (F) 3-Psycologist (F)	Female	83	5	Relative’s home/day centre	Daughter	Direct	Yes
	2	1-Pwd (M) 2-Daughter (F) 3-Grandchild (F)	Male	80	3	Own home	Daughter	Direct	No
	3	1-Pwd (F) 2- Husband (M) 3- Physiotherapist (M)	Female	64	1	Own home	Husband	Direct	Yes
**Total**	**22**	**56 Intervieweed** **41-Female** **15-Male**	**15 Females** **7 Males**	**Media: 79.23 Years old**	**Media: 5.68 Years**	**8 Own home** **2 Own home/hospital ward** **3 Own home/day centre** **2 Relative’s home/day centre** **5 Nursing home** **1 Dementia care home**	**8 Female relatives** **3 Male relatives** **6 Healthcare staff**	**14 Direct** **7 Indirect**	**14 Yes** **7 No**

A qualitative approach was taken. All interviews were face to face, conducted in a setting and at a time convenient to the interviewee. The interviews were conducted in the country’s mother language. The researchers conducting the interviews were part of each partner team in each partner country participating in the Palliare Project. Researchers experienced in working with vulnerable groups conducted the interviews. The same researcher/s conducted all the interviews in each country. The audio-recorded interviews focused on (a) what had worked well/currently works well, clarifying that we were referring to the strengths or positive aspects in daily life; (b) what had worked less well/currently works less well, also pointing out we were referring to the weaknesses or negative aspects in day-to-day situations. The qualitative technique selected was the open interview whose schedule comprised two main focuses that inspired the two open questions used as the starting point: (a) What works well about the experience or remarkable positive aspects in the advanced dementia caring process? and (b) What works not so well or remarkable negative aspects? As guidance, interviewers followed a list of topics corresponding with the categories used later in the analysis (Table 2). All the questions were open ended and designed to explore conversationally the lived experience of care and caring in daily life aspects, specifying that we were referring to their physical, psychological, social and also economic needs. Contextual information describing the environment of care was also recorded.

**Table 2.** Categories and subcategories for the first step of data analysis.

**Dementia Process/Adaptation**: Early symptom identification and first service encounters Assessment process Diagnostic disclosure/Getting a diagnosis Post-diagnostic support and appropiate interventions **Dementia Care. An Overview**: Patient’s emotions and behaviour/Mood Care environment/Housing and environment Help from other people Stress and emotions **Understand the caregiver’s role**: Dementia caregiver’s profile and effort involved Hours of care provided Type of support and how responsible they are Preparation for a caregiving role **Plan care for various stages of dementia** **Home adaptation for dementia patients/Managing at home** **Caregiving Essential Toolkit**: Communication Helping with Activities of Daily Living Daily routine Tips for specific activities of daily living Handling behaviour challenges Non-pharmacological approach Tools and tips Prevent the trigger, or modify the patient’s response/reduce posible harm Special tips for challeging behaviours: wandering, incontinece, repetitions, sundowning Late-stage dementia care/Transitioning to long-term care Acquisition of nursing skills Problems related to extended stay in bed Coexisting or new medical conditions End-of-life care decisions/End of life Grief and counselling Using trained attendants for dementia home care

Demographics and baseline characteristics are shown in Table 2.

The type of analysis selected for this study was thematic synthesis, whose purpose was a progressive theming to form a chain of reasoning. According to Thomas and Harden (2008), it is a method for identifying, analysing and reporting patterns (themes) within data [14]. It organizes and describes the data set in rich detail and interprets various aspects of the research topic. It can be used as a realist method that reports experience, meanings, and the reality of participants. It also examines the ways in which events, realities, meanings, experience, and other aspects affect the range of discourses The current analysis was based on responses from people with advanced dementia, family members and caregivers, both professional and non-professional, according to the context. To get to consensus and reach data validation, a double triangulation of qualitative data analysis was conducted through a four-step analysis procedure as shown in Figure 1.

Table 2 shows the categories and subcategories of data that were developed from the narrative accounts and field notes during the first step in the data analysis. In a second step, three trained researchers from the Spanish team (not involved directly in data collection) analysed all narrative accounts individually through the documents provided by each partner-team in the first step, then the three researchers arrived at common results about ‘what works well’ and ‘what works not so well’; that is to say, the positive and negative aspects were remarked. Later, in the third step, the cases were read back again to detect the themes that came up commonly across all the cases and their representativeness. In the fourth step, each partner-team was asked to provide a brief summary about what they considered that ‘works well’ and ‘what works not so well’ after having conducted the interviews to its cases, to validate the information previously provided and to try accordingly to overcome any loss of meaning through about the data having been translated into English.

## Results

### Each country prevalence and selected cases

Each partner-country team selected cases according to their experience in the field and the literature published [15] about the settings with the highest percentage of dementia population presented in Table 1 in the column named ‘Current setting of the advanced dementia care’.

### What works well and not so well in the experience of advanced dementia care

The positive experiences were associated with the particular outstanding organizational cultures associated with each country in terms of the type of services, units and professional workforce. The scope and type of provision of dementia care and support seemed to reflect the policy context in partner countries. For example, in southern Europe, policies tend to support family-integrated approaches whilst others tend to support individualized approaches to care and caregiving. There were many strong similarities in the negative experiences of care; that is to say, what works not so well within each country. The themes that came from the narrative accounts drew attention to the lack of care coordination and communication between staff and caregivers (interviewees used terms such as ‘coordination’, ‘organized’, ‘communication’), the lack of specific dementia education for the professional workforce/personnel and caregivers (interviewees used terms such as ‘training’, ‘education’) and the lack of investment in and funding for dementia care, and a lack of funding for professionals who specialized or had a special interest in dementia care, the investment in specific units and services, home adaptation and specific tools (interviewees used terms such as ‘funding’, ‘resources’, ‘workforce’).

Table 3 illustrates the strongest positive and negative experiences of dementia care in each of the countries.

**Table 3.** Strongest positive and negative aspects of dementia care in each country (Data analysis – second step).

Country	Remarkable Positve Aspects	Remarkable Negative Aspects
Scotland	Regular, sensitive and timely home visits from a profesional. Supportive professionals who are knowledgeable and caring.	Environments not dementia friendly (4–6 bedded wards, no garden, no quiet area, no signage, poor layout).
Slovenia	Dementia patients recognise community nurses’ support, they are familiar with them. They become an important professional and person in their lives.	Lack of organized and well-structured multidisciplinary workforce in the community fieldwork.
Portugal	There are special programmes for non-professional caregivers, training them to understand what dementia is. The family is invited to participate in the care process.	Lack of dissemination of the existing programmes for people with dementia in the health centres and at home.
Czech Republic	Continous support and information provided by the healthcare staff to the carers and the patients with dementia.	Poor communication or ineffective communication with relative in the last stages of the disease.
Finland	Person with dementia’s quality of life. If the quality of life of the family member is not good enough, then the quality of life of the affected person will be decreased.	Lack of training of education for family caregivers: family members had little or no training or education about dementia diseases. To get knowledge and support is necessary to understand dementia diseases. The ‘Memory Nurse’ has an important role between family members and official health and social services but more efforts are needed.
Sweden	Support from the different staff categories working in community health and social services.	Healthcare staff have a lack of empathy with the person with dementia. Some professionals do not know how to manage specific situations. Their training needs to be orientated to help people finding their own solutions through compassionate conversations.
Spain	Spanish culture promotes that people with dementia stay at home, surrounded by their loved ones. It seems to decrease the disease’s development, promoting the person’s routine and stabilization, makes him/her feel comfortable in his/her own and known environment.	Families and informal caregivers take care of the person with dementia in the best way possible or known by them, but sometimes they do not know how to act in difficult behavioural situations due to lack of knowledge about managing the difficult situations they have. There is a lack of follow-up to the caregiver and the person with dementia from the healthcare services.

#### The main themes in the European contexts explored

The main themes arising from the overall data analysis were: (a) *When dementia is not detected and diagnosed early, it limits the support strategies* that can be put in place in order for people to live well for longer. The experiences of many people who were presenting with early and mild symptoms of dementia were either diagnosed with other syndromes or in some cases the symptoms were considered part of the ‘normal’ ageing process. In some of the countries such as Spain, the Czech Republic, Finland, Slovenia and Portugal, *a lack of training* in recognizing and detecting dementia signs and symptoms was apparent. This has implications for identifying people with dementia early and putting in place support strategies. We also found that the healthcare, social and welfare systems of these countries tended to take a more active role when the clinical situation is advanced and most of their interventions are based on clinical decisions. It was in these later phases when the caregiver became visible to the systems. This has implications for carer support, involvement and partnership working. Opportunities are missed to support carers to support the person they are caring for, to support them both to live well for longer. Moreover, opportunities are missed to draw on the expertise of carers to work in partnership with the various care providers to plan and deliver care and support:

(Slovenia – Case 2/Social worker): At the primary level of health care, this topic is not recognized enough. … I feel that we, health care workers, don’t spend enough time on this … They just hear that the condition can’t be cured, Those who aren’t aggressive often just stay at home until the situation becomes acute and need to go to hospital..

(Spain – Case 1/Husband caregiver): I felt that she was more awkward with everything, losing some basic skills and of course losing memory … My daughters always told me that it wasn’t dementia … They thought it was a problem of the age … and the physicians even agreed with them in the beginning … but they were wrong.

(Scotland – Case/Husband caregiver): We waited nearly two years to get the diagnosis to say she’s got [Alzheimer’s].

*(b) There is a lack of coordination between service providers and a lack of care planning in dementia care*. There was evidence that showed that this directly impacted on the quality and continuity of care for the person with dementia and their family caregivers. It is important to remark that participants within the same countries, though in different care contexts, experienced quite *different care plans and interventions*. This appears to demonstrate a variety of approaches to advanced dementia care within countries:

(Finland – Case 1/Wife caregiver): Some tests were done at the beginning at home but there is no Care Plan … There’s something written, some papers in the drawer.

(Scotland – Case 1/Husband caregiver): She was then assessed at six-monthly intervals by the psychiatrist and was eventually given an official diagnosis of Alzheimer’s Disease approximately two years later.

(Scotland – Case 2/Hospital nurse): The transition from home to hospital is not good for people with dementia – very confusing – a ‘traumatic’ experience – they shouldn’t be admitted to hospital unless absolutely necessary.

(c) In many partner countries *there is an expectation, under* *pinned by cultural values about gender*, that female family caregivers will adopt the caring role, whilst in other countries there was a tendency for professionals to assume the caregiving role. We also found that in those countries with *family-oriented traditions* such as found in Southern Europe, government and public policies efforts focused on the ‘family’, particularly the ‘relative-carer’, to care for their relatives, whilst countries with individual-oriented traditions tended to focus their policies on providing resources and direct support for the person with dementia:

(Portugal – Case 4/Man with dementia): I’m happy to live with my daughter, despite my wife continue to supervise my daily activities and taking care of me.

(Spain – Case 3/Husband caregiver): I thought about the figure of a woman, someone who could live with us at home caring [for] my wife and I. When my wife will become worse, I have to be prepared. There must be someone that at the same time knows about how to care [for] a person with dementia [and] they need to be patient.

(Finland – Case 1/Man with dementia): I’m satisfied with my life, [my] wife makes food and takes care of me.

(d) A strong theme across almost all the countries was how the *sleeping pattern of the person with dementia affected the quality of life for the individual and their family carers*. This influenced the trajectory and type of care provided. When the person with dementia sleeps well, both they and their family caregivers maintain better physical and mental health and experience less stress and distress. However, when the person with dementia has disturbed sleep patterns, they experience an increase in distressed behaviours. Overnight sleep, rest and *privacy* were extremely important for carers, yet professionals and those not directly caring for the person with dementia rarely recognized the importance of this. The night-time experience of dementia appeared invisible to most professionals, and often to other members of the family and friends who were not primary caregivers. The sleeping pattern of the person with dementia and the impact that this was having on the primary caregivers was often an indicator of the extent to which they were managing to live well. Finding ways to support people with dementia to sleep well and supporting carers to get enough sleep, rest and privacy is extremely important if we are to enable the person with dementia and the family caregiver to live well for longer and maximize their quality of life:

(Portugal – Case 2/Daughter caregiver): When my father has any physical health problem, such as influenza or gastroenteritis, he always becomes more confused and aggressive and consequently has many nightmares. At such times it becomes more difficult for all to sleep and rest.

(Spain – Case 3/Daughter caregiver): Now, I have to leave my bedroom door open at night … I need to control that my father is sleeping.

(Czech Republic – Case 2/Daughter-in-law caregiver): She  increasingly performed night trips to the cabinet, did not recognize things; believed there was a toilet in his  bedroom for a toilet.

(e) Almost all of the *caregivers appreciated learning from professionals* about how best they can communicate with the person with dementia when their verbal skills began to diminish. This was particularly noticeable in those countries where there was less support for non-professional caregivers. In these countries there was a strong demand for more carer support from professionals. Professionals also remarked on the importance of *exchanging knowledge and experiences* about their daily situations *between different disciplines*. Use of resources, tips about how to face different situations, and communication skills in challenging scenarios were the most frequent topics:

(Spain – Case 3/Daughter caregiver): I have learnt it is better if you speak to them slowly with a lower tone of voice … I wish someone could have advised me all these things previously.

(Slovenia – Case 1/Community nurse): You have to take it slowly with them and repeat things many times, you ask repeatedly and give their words meaning, you have to let them tell you what they want … . This way, they gain new knowledge and learn about what their peers would do in a similar situation.

(Portugal – Case 1/Husband caregiver): The mental health nurse taught me some strategies to talk to my wife and it helps me to feel better. … She taught me how to stimulate my wife day by day and encouraged me to use music to improve her sense of well-being. It worked very well! She always became much calmer than without music therapy.

(f) Compassionate care, especially emphasizing the comfort and safety of those with dementia were highlighted during the interviews as well as including families in care planning. We found that in the southern European countries the main concern was to provide comfort to the person with dementia, whereas in the northern European countries the emphasis was more on the safety of the person, and tools and supports that could enable the person to continue to live safely:

(Finland – Case 3/Daughter caregiver): It is important that the day is always a good day for her … which means it will be good for us.

(Scotland – Case 3/Hospital nurse): For our residents and families to be happy, it makes such a difference when you work in partnership with the relatives; that makes a difference, they know their mum or their dad, so working in conjunction with them improves care.

(Slovenia – Case 2/Social worker): If you don’t have a big heart, you can’t be honest. And they know it if you’re not being honest. Whether you’re asking something just for the sake of it or whether you’re really interested, whether you really care.

(g) *The person with dementia preferred to remain in an environment familiar to them*, be connected to and sustain a social life and familiar routines. This seemed to allay some of the symptoms and enhance the quality of life. In all cases (22), it has been stated that the person’s quality of life depends on ‘being able to do something by herself/himself’ and ‘remaining to be in contact with her/his current social life’. In some cases, where the person with dementia had been moved from their home environment to another living environment (home or institutional), they experienced rapid disorientation and even isolation:

(Slovenia – Case 3/Man with dementia): I’m so far away but I want to die at home in the end [silence] … and it’s not so far, that moment. I want to be surrounded by my dear children, I’ve been alone for so long [silence] … if we could be close, live together [silence].

(Czech Republic – Case 1/Daughter caregiver): When they were introducing the percutaneous endoscopic gastrostomy (PEG) and I was waiting outside the room, I heard my mother crying. That’s when I’ve decided to transfer her home. I felt like they couldn’t treat her properly anymore. It produced no results and her condition got only worse.

(Sweden – Case 2/Husband caregiver): The staff are educated to care for persons with dementia. The goal is to reduce the burden for the family and to make it possible to live in the ordinary home as long as possible … . I think this is a good solution of our situation.

(h) *Experiences also suggested that there is a lack of qualified professionals who work in dementia care*. Moreover, we found that there was *a gap in evidence informed education on advanced dementia care* for both professional and non-professional caregivers:

(Slovenia Case 3/Hospital nurse): I read whatever I could find on this topic because every story with dementia is a little different. I know there’s plenty of information in books and [on the] Internet nowadays. Anyway, it was always connected with the person’s mental condition, so sometimes it was difficult to find solutions.

(Scotland – Case 3/Mental health nurse): If the staff are well trained, you know sometimes we hear if we could have more staff but I think it isn’t about more, it is about having trained staff, properly trained staff that know what they are doing.

(Sweden – Case 1/Daughter caregiver): Using trained attendants wasn’t always easy. Sometimes they could distract her and sometimes not.

## Discussion

### Comparison with other studies

We believe that this is the first published European case study of the advanced dementia care experience that includes the perspectives of people with advanced dementia, their professional and non-professional caregivers. The data provides unique insights into the current needs and viewpoints of people living with advanced dementia. This study is an attempt to fill the gap that other authors previously detected in the qualitative experience of the people living with and involved in dementia caring process across countries with cultural, demographic and policy differences [16]. Often the voices of people with advanced dementia are not heard due to the complexity of eliciting data from those with diminished verbal skills, their vulnerability and the ethical issues associated with their participation in research. However, we would argue that it is important to include people with advanced dementia so their voices and those of their carers are heard so as to inform our understanding of ageing and dementia [17].

In some studies, protective factors have been identified, including high levels of education [18], while in our study it is remarkable the presence of a clear protective factor, based on ‘living in her/his own current context’ instead of ‘family caring’. Some qualitative studies have exposed the importance of ‘family caring’, especially in Latin countries where the extended family is still found in society. However, having relatives caring for people with dementia could also be related to a society’s perception about its ineffective health system in which dementia care responsibility becomes a gap that families must overcome, due to the goverments’ lack of concern.

According to some studies, [19] people who develop Alzheimer’s disease typically retain insight into their condition at least in the mild to moderate stages of the disease. The perceptions about quality of life and the expressed needs of people living with dementia often differ from those of family caregivers [20]. This is something that we have also observed in those cases of people with advanced dementia who have verbal skills. We would argue that it is important that people living with dementia are kept involved in decision-making processes. As the condition progresses, responsibility for care and care-related decisions fall increasingly on professional and non-professional caregivers; however, creative approaches can be found to maintain some level of involvement of the person with advanced dementia in care-related decisions.

Dementia organizations have traditionally offered a source of information and support services more for caregivers than for people with advanced dementia [9]. However, such organizations appear particularly well established in Europe compared with other parts of the world in our study [21]. Despite this, the carers involved in this study expressed their need for more training and support. This study confirms previous research that showed that families receiving home support and people with Alzheimer’s disease receiving emotional support from local networks are more likely to be satisfied with the care experience [22]. This might suggest that the availability of generic and targeted support services to carers has room to grow and improve.

Our findings support other evidences which show that family members and other carers want access to detailed information about Alzheimer’s disease and opportunities to discuss the condition and treatment with professionals [16]. The participants in this study emphasized the need to discuss with professionals care planning to facilitate a coordinated approach to care. They specifically expressed a need for more home adaptation resources that would promote independent living for longer.

In fact, our findings demonstrated differences in the need for enhanced promotion of existing community services offered by healthcare organizations to raise awareness and increase accessibility in meeting needs across the continuum of the disease. Strong similarities have been found with the last Alzheimer Europe document published in 2014. The *Dementia in Europe Yearbook 2014* [9] provided equal outcomes, though they reviewed the support and care available to people with dementia after diagnosis in 30 European countries and focused only on the information provided by members of Alzheimer Europe in those countries through a questionnaire. Similarities with our study demonstrate that there are no clear national guidelines, though several local initiatives, and no official pathways; there is no unified, comprehensive and separated care for people with dementia; there is fragmented provision of services; the transitions among services are not seamless and there is poor coordination between services; there is no information sharing between health and social systems; there is a lack of integrated models of care; and the system is complex.

Moreover, during the data analysis, discussions among the seven partner teams on the variations in the results depending on their health systems’ organization and policies regarding dementia care in their countries and regions took place. As part of those discussions, researchers highlighted that policies developed and implemented in relation to dementia care and palliative care for people with dementia differ greatly across the partner countries. Scotland particularly has more specific dementia policies that include national dementia strategies and workforce development frameworks; this is in contrast to the Czech Republic where policies to support dementia care are very much in their infancy. In Slovenia the presence of community staff permits a connection between the demands and the offer of dementia care although there is still a lack of an organized and well-structured multidisciplinary workforce. In Portugal there are a lack of Dementia Care Implementation programmes as the resources coming from the defined strategies do not count with implementation strategies for dementia population, they are still in a very descriptive programme phase. Finland’s healthcare system is aware of the importance of caring for people with dementia and their main caregivers individually; however, there are not enough resources to meet all the demands of both main actors. There is a similar scenario in Spain, where main caregivers play an important role in caring for people with dementia as community healthcare staff can not assume the tremendous demand associated with dementia community care. On the contrary, Sweden counts with a multidisciplinary healthcare staff that addresses dementia care in the community from a theoretical perspective, but dementia education should meet the emotional intelligence demands of people with dementia and their loved ones.

### Strengths and limitations

The case study method allowed us to begin mapping the complex picture of advanced dementia care across European contexts. A strength in the case study design was the ability to see the caring situation and understand experiences from multiple perspectives: the person with advanced dementia, the professional and the non-professional caregiver. The combination of semi-structured interviews and observational design allowed an in-depth exploration of real-life conditions and experiences. The design also facilitated insight into the experiences of living with and caring for people with dementia in caring contexts considered typical for people with advanced dementia in seven European countries.

A limitation of the case study is that it is vulnerable to the changes experienced by societies and therefore a recommendation for future replication of the same research design should be developed. In spite of this we did not identify different evidences compared with previous studies and recommendations published until this moment.

An inherent risk in international studies is that some meanings from interview data can be lost in translation. We offset this risk through our four-step process of analysis outlined earlier.

As might be expected with international projects, several factors impinged on data collection plans. In some countries such as Sweden and Slovenia we were not able to obtain the ethical permissions to collect data directly from individuals or families with advanced dementia, as originally intended. However, we intended to compromise and build case studies from other key informants to comply with the recommendations of the National Ethics Committees, meeting all the ethical requirements. We found that some families were protective of their relatives with dementia, preferring them not to have a direct voice in the interview process: a request we respected.

A further challenge was identification of individuals with ‘advanced dementia’ due to general lack of clarity concerning diagnosis and the progression of dementia. This was compounded by a lack of dementia services in some countries and inconsistencies in service provision.

It is important to remark at this point that law and policy regulations related to dementia have been developed to different extents in the seven countries participating and also in the rest of Europe; and at the same time especially emphasizing in each country the distinct aspects of the development of dementia organizations, their workforce and resources.

This study did not include young people or people with other pre-existing conditions such as intellectual disability who had advanced dementia. Future studies could concentrate efforts on exploring the experience of advanced dementia more widely across Europe, considering other context samples, though it is not a limitation itself of the case study method.

## Conclusions

The evidence presented in this study points to the following priorities for action: (a) *Prevention* of potential risks, errors, complications and harmful events for the person affected and caregivers, supporting people to make healthy choices. (b) *Support after diagnosis* with an appropriate and personalized care plan, integrated and multidisciplinary. (c) *High-* *quality and compassionate care* everywhere, in all the contexts, providing people with flexible, appropriate and timely evidence-based cares monitored by skilled staff whether at home, in hospital or in a care home. (d) *Greater personal control*, enabling those with dementia and their carers to exert control over their care and over their lives even in advanced stages, including end of life. (e) *Dementia education and training* for health and social care staff and non-professional carers, who should be aware of the signs of dementia and how best to support people with the condition, their families and carers, consequently achieving dementia-friendly communities and societies. (f) *Social i* *mpact r* *esearch* with better data and evidence, including people from different fields to improve the availability and quality of data on dementia care and support.

## Future implications

To achieve those priorities, the Palliare Project is designing education through an academy that makes use of modern technology, communication and networking to support learning in a virtual environment through membership of a facilitated virtual international community of practice (Dementia Palliare Community of Practice [17]) based on experiential learning. It will also design three interprofessional higher education modules on advanced dementia care to be developed in different European higher education organizations and consequently in different languages, the content of which will reflect the main themes arising from the case studies. The empirical data gathered on the experience of advanced dementia care in each country will inform the development of the European Best Practice Statement (BPS) just created [16] and the interprofessional modules on advanced dementia care. The Palliare BPS has been created as a tool to guide and address educational deficiencies in interprofessional dementia education based on currently available evidence. The vision of the Palliare Project research team consists in creating a positive society where people can live well with dementia, respecting the context and culture, with a greater global collaboration and leadership to improve the lives of those affected.

## Paper context

Systematic Review: All relevant information about research initiatives focusing on qualitative methodology, specifically on applied Case Study Method in Europe. Interpretation: Mapping the Experience of Advanced Dementia Care in Europe is crucial towards improving the quality of life and care conditions in the context of those affected. Future directions: Prospective studies are needed in other European contexts to keep exploring what works well in dementia care and also to work further on the negative aspects.
